# Elevated Plasma Complement C1q Levels Contribute to a Poor Prognosis After Acute Primary Intracerebral Hemorrhage: A Prospective Cohort Study

**DOI:** 10.3389/fimmu.2022.920754

**Published:** 2022-06-23

**Authors:** Zefan Wang, Xiaoyu Wu, Tian Yan, Ming Liu, Wenhua Yu, Quan Du, Wei Hu, Yongke Zheng, Zuyong Zhang, Keyi Wang, Xiaoqiao Dong

**Affiliations:** ^1^ The Fourth School of Clinical Medicine, Zhejiang Chinese Medical University, Hangzhou, China; ^2^ Department of Neurosurgery, Affiliated Hangzhou First People’s Hospital, Zhejiang University School of Medicine, Hangzhou, China; ^3^ Department of Intensive Care Unit, Affiliated Hangzhou First People’s Hospital, Zhejiang University School of Medicine, Hangzhou, China; ^4^ Department of Neurosurgery, Xixi Hospital Affiliated to Zhejiang Chinese Medical University, Hangzhou, China; ^5^ Central Laboratory, Affiliated Hangzhou First People’s Hospital, Zhejiang University School of Medicine, Hangzhou, China

**Keywords:** intracerebral hemorrhage, complement component 1q, mechanism, prognosis, severity

## Abstract

**Objective:**

The complement cascade is activated early following intracerebral hemorrhage (ICH) and causes acute brain injury. We intended to explore the effects of plasma complement component 1q (C1q) levels on hemorrhagic severity and functional outcome in ICH patients.

**Methods:**

In this prospective cohort study, we measured the plasma C1q levels of 101 ICH patients and 101 healthy controls. The Glasgow Coma Scale (GCS) score and hematoma volume were used to assess the ICH severity. Poor prognosis was referred to as a Glasgow Outcome Scale (GOS) score of 1-3 at three months following a stroke. A multivariate logistic regression model was configured to determine the independent relation of plasma C1q levels to severity and poor prognosis. Under receiver operating characteristic (ROC) curve, prognostic capability of plasma C1q levels was evaluated.

**Results:**

There was a significant elevation of plasma C1q levels in patients, as compared to controls [median (percentiles 25th-75th), 225.04 mg/l (156.10-280.15 mg/l) versus 88.18 mg/l (70.12-117.69 mg/l); P<0.001]. Plasma C1q levels of patients were independently related to GCS score (t =-3.281, P=0.001) and hematoma volume (t = 2.401, P=0.018), and were highly correlated with the GOS score at 3 months post-stroke (r=-0.658, P<0.001). Plasma C1q levels were obviously higher in poor prognosis patients than in other remainders (median percentiles 25th-75th), 278.40 mg/l (213.81-340.05 mg/l) versus 174.69 mg/l (141.21-239.93 mg/l); P<0.001). Under the ROC curve, plasma C1q levels significantly discriminated the development of poor prognosis (area under ROC curve 0.795; 95% confidence interval, 0.703–0.869; P<0.001). Using maximum Youden method, plasma C1q levels > 270.11 mg/l distinguished patients at risk of poor prognosis at 3 months with 56.52% sensitivity and 94.55% specificity. Meanwhile, the prognostic predictive ability of plasma C1q levels was equivalent to those of GCS score and hematoma volume (both P>0.05). Moreover, plasma C1q levels > 270.11 mg/l independently predicted a poor prognosis at 3 months (odds ratio, 4.821; 95% confidence interval, 1.211-19.200; P=0.026).

**Conclusion:**

Plasma C1q levels are closely related to the illness severity and poor prognosis of ICH at 3 months. Hence, complement C1q may play an important role in acute brain injury after ICH and plasma C1q may represent a promising prognostic predictor of ICH.

## Introduction

Intracerebral hemorrhage (ICH) belongs to severe cerebrovascular diseases and is characterized by high mortality and significant morbidity in survivors (1). The Glasgow Coma Scale (GCS) and bleeding size are conventionally recorded for assessing ICH severity and predicting clinical outcome (2). During the early phase after ICH, activation of the complement cascade is implemented *via* classical, alternative, or lectin pathways and contributes to inflammatory response, neuronal death, brain edema, and damaged blood-brain barrier, thereby participating in a secondary brain injury after ICH (3). However, the role of complement cascade in ICH remains unclear and subsequently understanding its role may help to elucidate possible interventions to lessen brain injury and further improve neurologic recovery following ICH.

Complement component 1q (C1q) is the first subcomponent of the C1 complex, which participates in the classical pathway of complement activation (4). By binding to either immunoglobulin or non-immunoglobulin activators of the complement system, C1q plays a crucial role in adaptive and innate immunity (5). Clearly, C1q can be synthesized in microglia of the mouse brain (6) and neurons of aged rhesus macaque dorsolateral prefrontal cortex (7). Interestingly, there was a significant up-regulation of C1q expression in rat brain microglia after transient global cerebral ischemia (8) and traumatized brain tissues of humans (9). Alternatively, increased serum C1q levels after acute ischemic stroke were positively correlated with neurological injury severity and infarct size of patients (10, 11). Moreover, in a recent clinical study, elevated serum C1q levels were strongly correlated with traumatic severity and independently associated with a 6-month poor clinical outcome in patients with traumatic brain injury (12). Therefore, it is speculated that C1q might be a potential biochemical marker of acute brain injury. However, to the best of my knowledge, C1q levels in peripheral blood of ICH humans have not been studied. In the current study, we evaluated whether plasma C1q levels are associated with illness severity and a 3-month prognosis in patients with ICH, and further determined the clinical utility of plasma C1q as a potential predictor of severity and clinical outcome of human ICH.

## Materials and Method

### Study Population

In this prospective cohort study, we included all patients with first-ever acute spontaneous ICH admitted to Affiliated Hangzhou First People’s Hospital, Zhejiang University School of Medicine (Hangzhou, China) between May 2020 and September 2021. In addition, all patients were hospitalized within 24 hours post-stroke and their hematomas were treated conservatively. The exclusion criteria were: 1) age under 18 years; 2) ICH resulting from traumatic brain injury, venous sinus thrombosis, intracranial aneurysms, moyamoya’s disease, arteriovenous malformation, ischemic stroke, or intracranial tumors; 3) previous neurological diseases, such as hemorrhagic or ischemic stroke, intracranial tumors and severe head trauma; and 4) other specific diseases, such as severe infections within the past four weeks, autoimmune diseases, and previously diagnosed malignancies. Meanwhile, a group of healthy individuals were selected as controls. The current study was conducted in accordance with the ethical guidelines of the Declaration of Helsinki, and approval for the study protocol was acquired from the Institutional Review Committee at Affiliated Hangzhou First People’s Hospital, Zhejiang University School of Medicine (Opinion number (2020): Medical Ethics Review No. (058)-01). The informed consent for participating in this study was signed by the legal representatives of patients and controls themselves.

### Data Collection and Immune Analysis

Relevant information such as, demographics, vascular risk factors, medical history, medications, clinical scores, vital signs, imaging parameters, location of hematoma, hematoma volume and intraventricular hemorrhage was collected and stroke severity was assessed with GCS at admission. The hematoma volume was measured *via* the ABC/2 method (13). The functional outcome was evaluated by the Glasgow Outcome Scale (GOS). Patients with GOS score of 1-3 at 3 months after the stroke were considered to have an adverse outcome (14).

Peripheral blood samples of patients and controls were collected on admission and at admission into study, respectively. Within 30 minutes, blood samples were centrifuged at 3000 g for 10 minutes, and afterwards plasma samples were stored at -80°C for final analysis. Plasma C1q levels were quantitatively measured utilizing the enzyme-linked immunosorbent assay with a commercially available kit (Shanghai Kexing Trading Co., Ltd, Shanghai, China) according to the manufacturer’s instructions. Every 3 months, the quantifications were performed, in duplicate, by the same technician inaccessible to clinical data and the two measurements were averaged for final analysis.

### Statistical Analysis

Statistical analyses were carried out with Statistical Package for Social Sciences Version 25.0. (IBM Corp., Armonk, NY, USA) and MedCalc 9.6.4.0 (MedCalc Software, Mariakel, Belgium). We plotted the figures *via* GraphPad Prism version 9.0 (GraphPad Software Inc., La Jolla, CA, USA). Qualitative data were presented as counts (percentages). The Kolmogorov–Smirnov test was conducted to determine normal distribution of quantitative variables. In this study, other quantitative variables, except for age, were not normally distributed. Non-normally distributed data were summarized as medians (lower and upper quartiles) and normally distributed data were reported as means ± standard deviations. Where appropriate, intergroup differences of data were analyzed by the χ2 test, Fisher’s exact test, unpaired t-tests, or the Mann-Whitney U tests. Using the Kruskal–Wallis H test, plasma C1q levels were compared among multiple groups, which were divided using GOS scores. The Spearman correlation coefficient was carried to analyze bivariate correlations and thereafter the variables, which were found to be significantly correlated with plasma C1q levels (P<0.05), were forced into a multivariate linear regression model. In order to determine independently associated variables with poor prognosis, a binary logistic regression model was built which adjusted for other significant variables (P<0.05) in univariate analysis. Odds ratios (ORs) and 95% confidence intervals (CIs) were reported for showing associations. Area under receiver operating characteristic (ROC) curve (AUC) and the corresponding 95% CI were estimated for assessing prognostic predictive values. A P value of <0.05 indicated statistical significance.

## Results

### Patient Selection and Subject Characteristics

**Figure 1:**
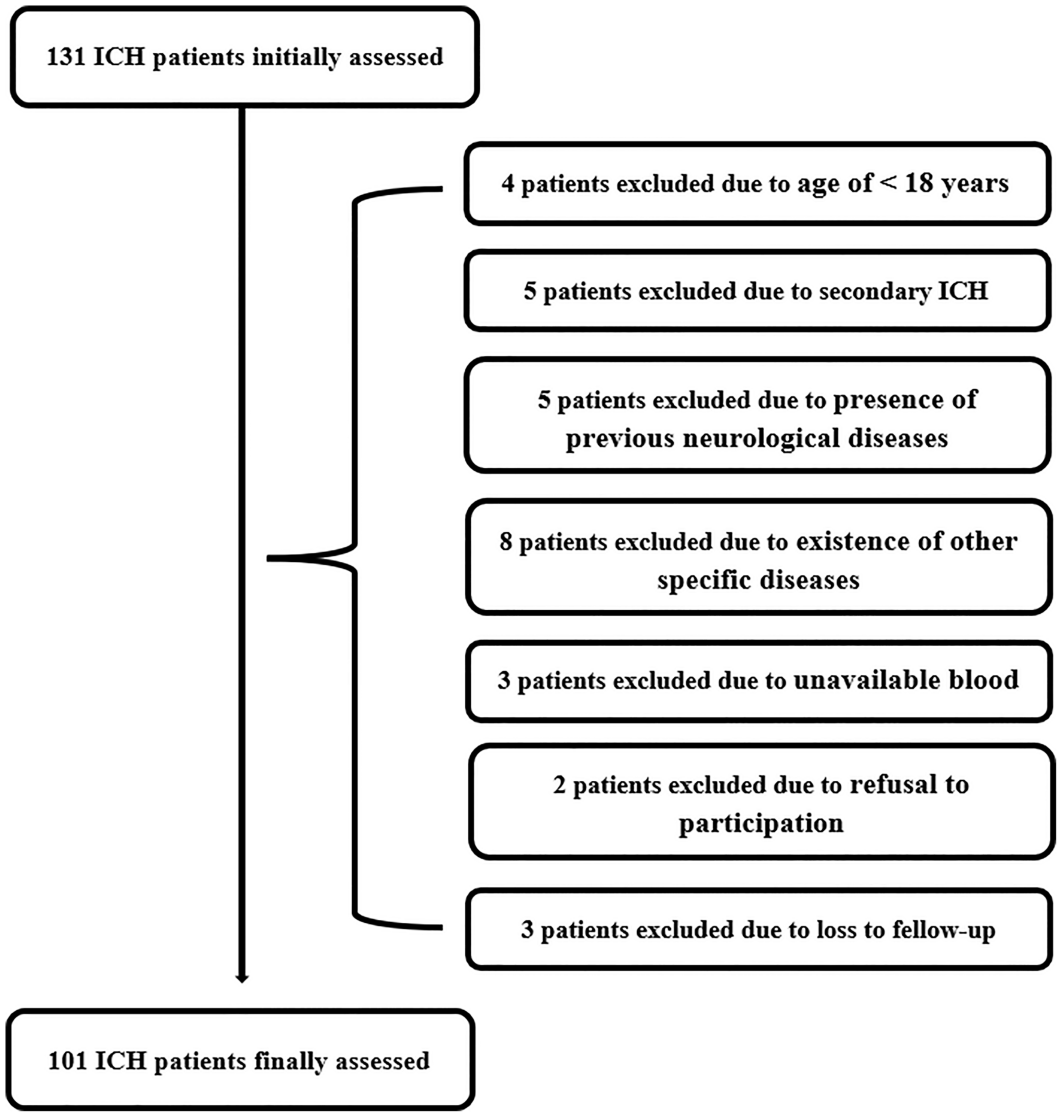
Flow chart of selecting eligible patients with acute spontaneous intracerebral hemorrhage. Initially, 131 intracerebral hemorrhage patients were assessed; thereafter, we excluded 30 patients; and ultimately, 101 patients were recruited. ICH indicates intracerebral hemorrhage.

As shown in **Figure 1**, we initially assessed 131 patients with first-ever acute spontaneous ICH who were hospitalized within 24 h post-stroke and whose hematomas were treated conservatively. Thereafter, we excluded thirty patients according to exclusion criteria and ultimately, 101 patients with acute ICH were recruited. In addition, a total of 101 healthy individuals were recruited as controls. In **Table 1**, as compared to controls, patients had similar mean age, proportion of males, current smokers, and alcohol drinkers, as well as were more likely to have significantly elevated percentages of hypertension and diabetes mellitus.

**Table 1:** Comparisons of demographic data and vascular risk factors between controls and patients with acute intracerebral hemorrhage.

	Patients	Control	P value
Age (years) Sex (male/female) Hypertension Diabetes mellitus Current smoking Alcohol consumption	61.7 ± 12.2 59/42 61 (60.4%) 15 (14.8%) 21 (20.8%) 26 (25.7%)	59.5 ± 14.3 56/45 0 0 19 (18.8%) 22 (21.8%)	0.915 0.670 < 0.001 < 0.001 0.724 0.508

Quantitative data were reported as medians with 25th–75th percentiles or the mean ± standard deviation as appropriate. Qualitative data were presented as counts (proportions). Intergroup comparisons of various variables were performed using the χ2 test or Fisher’s exact test for qualitative data, and Mann–Whitney U-test for quantitative data.

This group of ICH patients (59 males and 42 females) were 32 to 89 years of age (mean, 61.7 years; standard deviation, 12.2 years). In total, 21 patients were cigarette smokers and 26 patients were alcohol consumers. A total of 61 patients suffered from hypertension and 15 patients were inflicted with diabetes mellitus. The patients were hospitalized from 0.5 to 24 hours after the onset of stroke symptoms (median, 6.5 hours; lower and upper quartiles, 3.9-11.6 h), with their blood samples drawn from 1.0 to 25.0 h (median, 7.0 h; lower and upper quartiles, 4.0-12.0 h) after the stroke onset. Admission GCS score ranged from 4-15 (median, 13; lower and upper quartiles, 8-14). Hematomas were located at infratentorial cavity in 18 cases. Intraventricular bleedings were revealed in 14 cases. Hematoma volume ranged from 2.5 to 64.3 ml (median, 14.5 ml; lower and upper quartiles, 8.1-25.1 ml). Using non-invasive techniques, it was revealed that systolic arterial pressure and diastolic arterial pressure ranged from 100 to 195 mmHg (median, 155 mmHg; lower and upper quartiles, 135.5-167 mmHg) and from 55 to 122 mmHg (median, 91 mmHg; lower and upper quartiles, 80.5-100 mmHg), respectively. Blood leucocyte count ranged from 3.4 to 24.2×10^9^/l (median, 8.9×10^9^/l; lower and upper quartiles, 6.8-11.15×10^9^/l), and serum glucose levels ranged from 2.5 to 18.9 mmol/l (median, 6.6 mmol/l; lower and upper quartiles, 5.35-8.35 mmol/l). Post-stroke 3-month GOS scores 1, 2, 3, 4 and 5 were revealed in 6, 11, 29, 11 and 44 patients respectively, with a median value of 4 at GOS score (range, 1-5; lower and upper quartiles, 3-5). In total, 46 patients had a poor outcome at 3 months (GOS score 1-3).

### Change of Plasma C1q Levels and Its Correlation With Bleeding Severity

**Figure 2:**
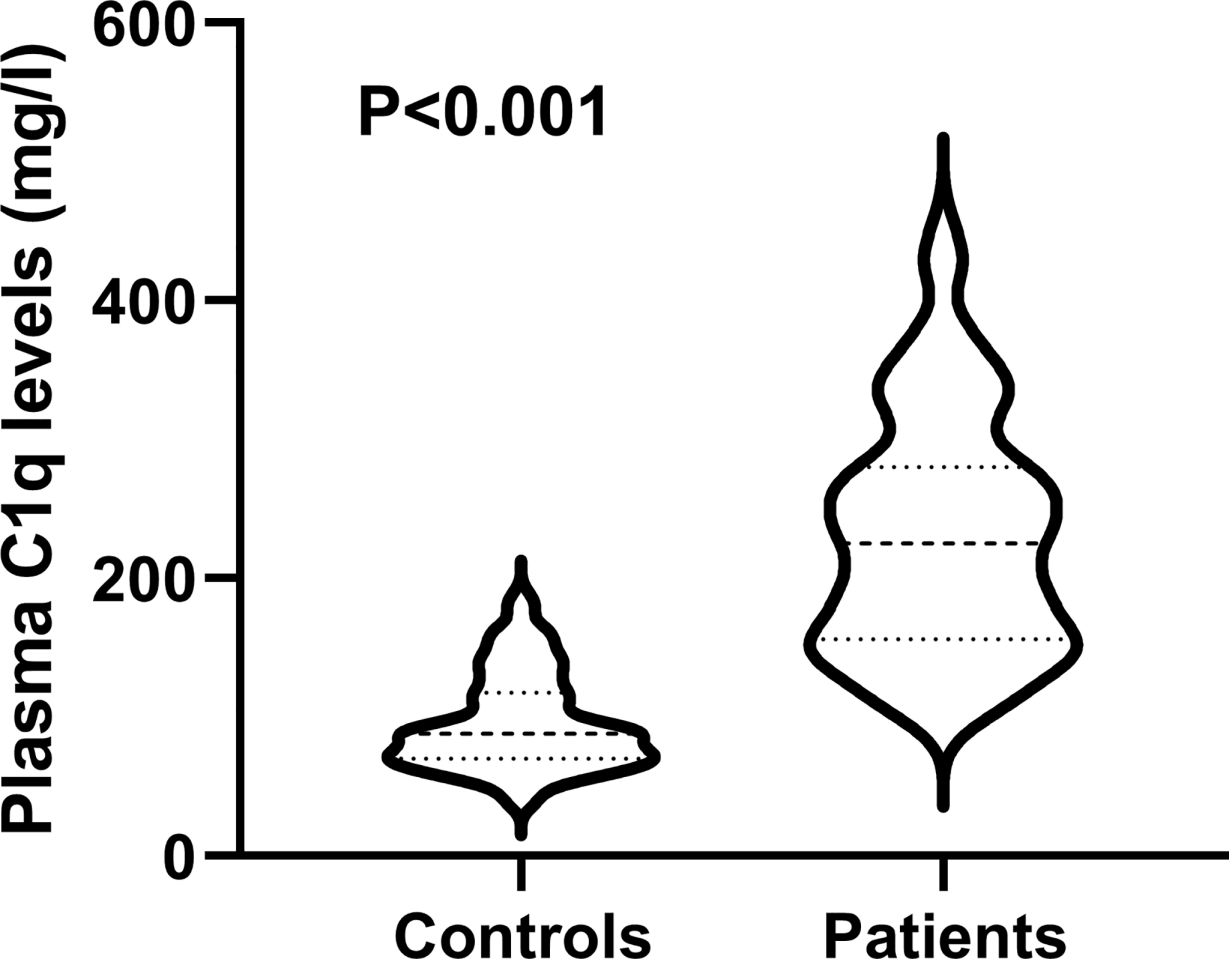
Differences in terms of plasma C1q levels between healthy controls and patients with intracerebral hemorrhage. Plasma C1q levels were reported as median (upper-lower quartiles). Plasma C1q levels were significantly higher in patients than in healthy controls using the Mann–Whitney U test (P < 0.001).

**Figure 3:**
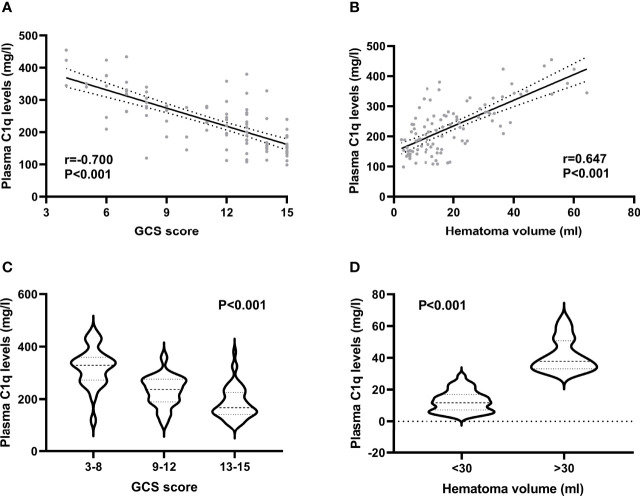
Relationship between plasma C1q levels and hemorrhagic severity after acute intracerebral hemorrhage. **(A)** Relationship between plasma C1q levels and Glasgow Coma Scale score after acute intracerebral hemorrhage. Plasma C1q levels were tightly correlated with Glasgow Coma Scale score following stroke using the Spearman’s correlation coefficient (P < 0.001). GCS indicates Glasgow Coma Scale. **(B)** Differences of plasma C1q levels by hematoma volume after acute intracerebral hemorrhage. There were substantial differences in terms of plasma C1q levels after stroke among multiple groups using the Kruskal−Wallis test (P < 0.001). **(C)** Comparisons of plasma C1q levels across severity grade among patients with acute intracerebral hemorrhage. Significant differences of plasma C1q levels existed after stroke among multiple groups using the Kruskal−Wallis test (P < 0.001). GCS indicates Glasgow Coma Scale. **(D)** Relation of plasma C1q levels to hematoma volume after acute intracerebral hemorrhage. Plasma C1q levels were closely correlated with hematoma volume after stroke using the Spearman’s correlation coefficient (P < 0.001).

Plasma C1q levels in patients with ICH were significantly higher than those in healthy controls (**Figure 2**). In order to discern the relationship between plasma C1q levels and hemorrhagic severity indicated by GCS score and hematoma volume in this cohort of ICH patients, both GCS score and hematoma volume were considered as not only categorical, but also continuous variables. Subsequently, it was revealed that (1) plasma C1q levels were substantially negatively related to GCS score when the GCS score was regarded as a quantitative variable (P<0.001) (**Figure 3A**); (2) when hematoma volume was shown as a continuous variable, plasma C1q levels tended to increase significantly with rising hematoma volume (P<0.001) (**Figure 3B**); (3) plasma C1q levels were markedly higher in patients with GCS scores of 3-8 than in those with GCS scores of 9-12 (P<0.001), as well as in patients with GCS scores of 9-12 than in those with GCS scores of 13-15 (P<0.001) (**Figure 3C**); and (4) compared to patients with hematoma volume less than 30 ml, those presenting with hematoma volume more than 30 ml had significantly elevated plasma C1q levels (P<0.001) (**Figure 3D**).

**Table 2** shows that plasma C1q levels were closely related to GCS score, hematoma volume, intraventricular hemorrhage, and blood glucose level. The above-mentioned significantly correlated variables were forced into the multivariate linear regression model and thereafter, it was confirmed that plasma C1q levels had an independent correlation with GCS score and hematoma volume (**Table 3**).

**Table 2:** Bivariate correlation analysis between plasma C1q levels and other variables in 101 intracerebral hemorrhage patients.

Components	r	P value
Age (years) Sex (male/female) Hypertension Diabetes mellitus Current smoking Alcohol consumption Admission time (h) Blood-collection time (h) Systolic arterial pressure (mmHg) Diastolic arterial pressure (mmHg) infratentorial hemorrhage Intraventricular hemorrhage Glasgow Coma Scale score Hematoma volume (ml) Blood leucocyte count (×10^9^/l) Plasma glucose levels (mmol/l) Plasma potassium level (mmol/l)	0.190 -0.035 0.087 0.086 0.159 -0.010 -0.145 -0.136 -0.062 -0.015 -0.020 0.355 -0.700 0.647 0.150 0.199 -0.075	0.057 0.730 0.384 0.393 0.112 0.920 0.147 0.175 0.539 0.881 0.846 *< 0.001 *< 0.001 *< 0.001 0.135 *0.046 0.457

Correlations were done using Spearman Correlation Coefficient in Intracerebral Hemorrhage. The asterisk indicates statistical significance (*P < 0.05).

**Table 3:** Multivariate linear regression analysis between elevated plasma C1q levels and other variables.

Components	t	P value
Intraventricular hemorrhage Glasgow Coma Scale score Hematoma volume (ml) Plasma glucose levels (mmol/l)	0.012 -3.281 2.401 -0.055	0.990 *0.001 *0.018 0.956

Correlations was presented using multivariate linear regression model. The asterisk indicates statistical significance (*P < 0.05).

### Relationship Between Plasma C1q Levels and Neurologic Functional Outcome

**Figure 4:**
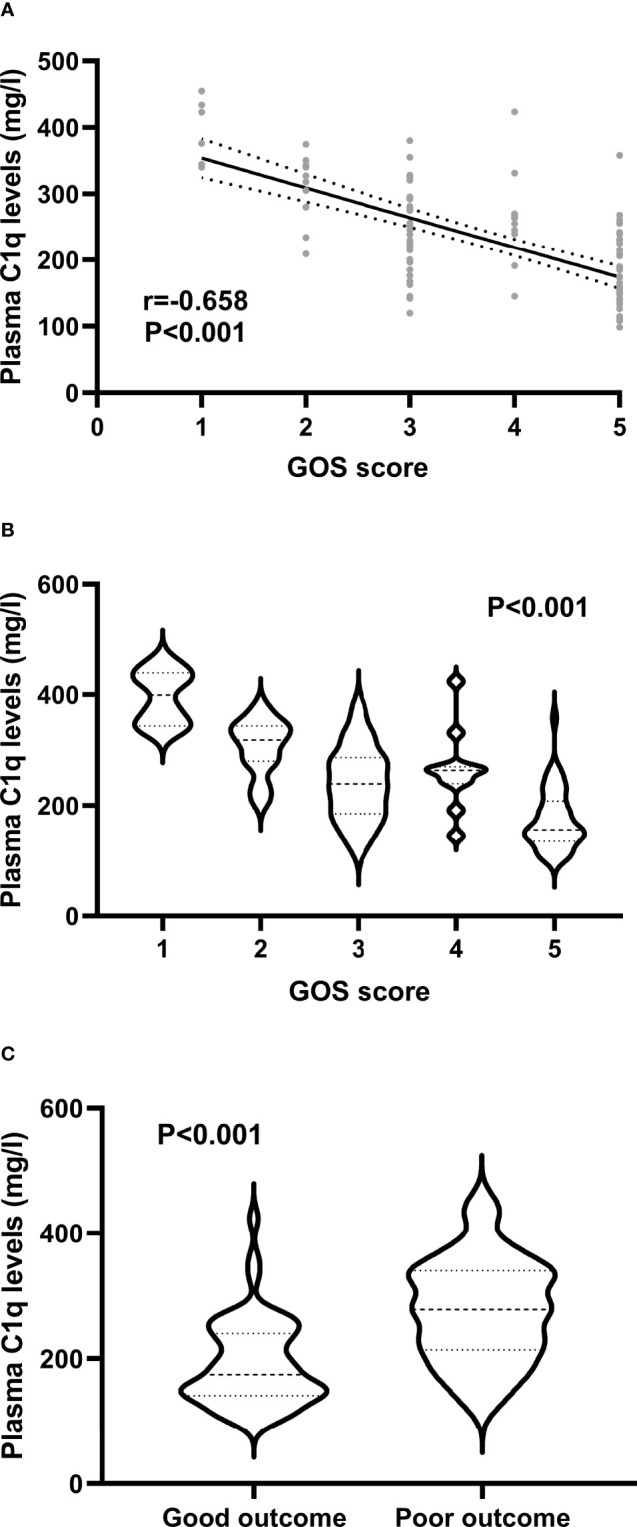
Association of plasma C1q levels with prognosis at 3 months after acute intracerebral hemorrhage. **(A)** Association of plasma C1q levels with Glasgow Outcome Scale score after intracerebral hemorrhage. Plasma C1q levels were highly correlated with Glasgow Outcome Scale score after stroke using the Spearman’s correlation coefficient (P < 0.001). GOS means Glasgow Outcome Scale. **(B)** Differences of plasma C1q levels by Glasgow Outcome Scale score after acute intracerebral hemorrhage. There were substantial differences in terms of plasma C1q levels after stroke among multiple groups using the Kruskal-Wallis test (P < 0.001). GOS denotes Glasgow Outcome Scale. **(C)** Differences of plasma C1q levels between patients with Glasgow Outcome Scale score 1-3 and those with Glasgow Outcome Scale score 4-5 at 3 months after stroke. Plasma C1q levels were markedly raised in patients with Glasgow Outcome Scale score 1-3, as compared to those presenting with score 4-5 at 3 months after stroke using the Mann–Whitney U test (P < 0.001).

**Figure 5:**
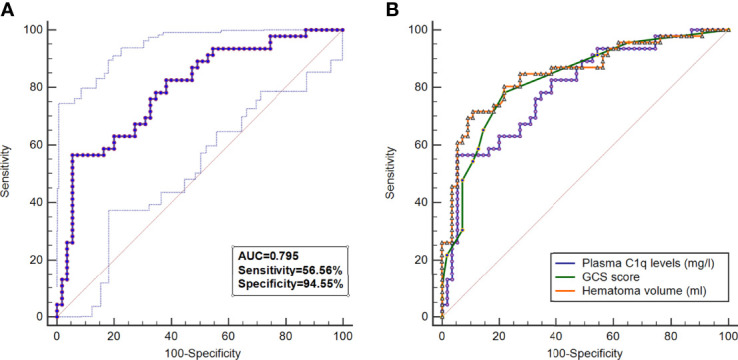
Predictive value of plasma C1q levels for poor outcome at 3 months after intracerebral hemorrhage. **(A)** Under the receiver operating characteristic curve, plasma C1q levels significantly discriminated development of poor prognosis (area under curve 0.795; 95% confidence interval, 0.703–0.869; P < 0.001). Using maximum Youden method, plasma C1q levels > 270.11 mg/l distinguished patients at risk of 3-month poor prognosis with 56.52% sensitivity and 94.55% specificity. AUC denotes area under curve. **(B)** Comparison of discriminatory capability with respect to plasma complement component 1q levels, Glasgow Coma Scale score and hematoma volume for 3-month poor prognosis following acute intracerebral hemorrhage under receiver operating characteristic curve. Poor prognosis was defined as Glasgow Outcome Scale score 1-3. Prognostic predictive ability of plasma Complement component 1q levels (area under curve, 0.795; 95% confidence interval, 0.703-0.869) was similar to those of Glasgow Coma Scale score (area under curve, 0.827; 95% confidence interval, 0.739-0.895; P=0.425) and hematoma volume (area under curve, 0.850; 95% confidence interval, 0.765-0.913; P=0.205). GCS indicates Glasgow Coma Scale.

A total of 46 (45.5%) patients had a poor prognosis (GOS scores 1-3) at 3 months after ICH and **Figure 4A** shows that plasma C1q levels tended to decrease significantly with the increase in GOS scores. Also, plasma C1q levels were significantly different among subgroups with different GOS scores (**Figure 4B**). In addition, patients with GOS scores of 1-3 had significantly higher plasma C1q levels than other remainders (**Figure 4C**). According to the ROC curve in **Figure 5A**, plasma C1q levels significantly predicted the development of poor prognosis at 3 months post-stroke with an AUC of 0.795 (95% CI, 0.703–0.869); and using the maximum Youden method, plasma C1q levels > 270.11 mg/l distinguished patients at risk of 3-month poor prognosis with 56.52% sensitivity and 94.55% specificity. Meanwhile, the prognostic predictive capability of plasma C1q levels was in range of GCS score and hematoma volume (both P>0.05) (**Figure 5B**).

As shown in **Table 4**, compared with patients with good prognosis, those with poor prognosis had substantially lower GCS score, had a significantly higher percentage of intraventricular hemorrhage, exhibited markedly higher hematoma volume, and displayed profoundly higher plasma C1q levels and blood glucose levels. Multivariate analysis demonstrated that hematoma volume and plasma C1q levels were the two independent predictors of poor prognosis at 3 months after hemorrhagic stroke (**Table 5**).

**Table 4:** Demographic, Clinical, Radiological and Biochemical Factors for 3-month Poor Outcome After Acute Intracerebral Hemorrhage.

Components	Poor outcome	Good outcome	P value
Number Age (years) Sex (male/female) Hypertension Diabetes mellitus Current smoking Alcohol consumption Admission time (h) Blood-collection time (h) Systolic arterial pressure (mmHg) Diastolic arterial pressure (mmHg) Infratentorial hemorrhage Intraventricular hemorrhage Glasgow Coma Scale score Hematoma volume (ml) Blood leucocyte count (×10^9^/l) Plasma glucose levels (mmol/l) Plasma potassium level (mmol/l) Plasma C1q > 270.11 mg/l	46 (45.5%) 62.0 ± 12.9 29/17 29 (63.0%) 7 (15.2%) 8 (17.4%) 9 (19.6%) 7.1 (4.0-11.9) 7.5 (4.0-12.2) 153 (129-163) 92 (78-100) 6 (13.0%) 12 (26.1%) 9 (7-12) 24.6 (15.7-37.2) 9.2 (6.9-11.5) 7.4 (5.7-9.0) 3.64 (3.39-3.90) 26 (56.5%)	55 (54.5%) 61.5 ± 11.6 32/23 32 (58.2%) 8 (14.5%) 13 (23.6%) 17 (30.9%) 6.0 (3.9-11.6) 6.0 (4.0-12.0) 157 (141-171) 90 (82-101) 12 (21.8%) 2 (3.6%) 13 (13-15) 10.7 (6.0-14.7) 8.4 (6.5-10.8) 6.2 (5.1-8.0) 3.62 (3.43-3.87) 4 (7.3%)	0.702 0.619 0.619 0.925 0.441 0.194 0.542 0.498 0.064 0.581 0.251 *0.001 *<0.001 *<0.001 0.127 *0.042 0.959 *<0.001

Quantitative data were reported as medians with 25th-75th percentiles or the mean ± standard deviation as appropriate. Qualitative data were presented as counts (proportions). Intergroup comparisons of various variables were performed using the χ^2^ test or Fisher’s exact test for qualitative data, and Mann-Whitney U-test for quantitative data. Glasgow Outcome Scale score of 1–3 was designated as poor outcome. The asterisk indicates statistical significance (*P < 0.05).

**Table 5:** Multivariate logistic regression analysis for risk factors of poor 3-month prognosis in acute intracerebral hemorrhage.

Variables	Odds ratio (95% confidence interval)	P value
Glasgow Coma Scale score Hematoma volume (ml) Intraventricular hemorrhage Plasma C1q > 270.11 mg/l Plasma glucose levels (mmol/l)	0.967 (0.716-1.306) 1.114 (1.021-1.216) 1.243 (0.161-9.590) 4.821 (1.211-19.200) 0.997 (0.793-1.254)	0.825 *0.015 0.835 *0.026 0.979

Results were showed as odds ratio (95% confidence interval) according to the binary logistic regression analysis. The asterisk indicates statistical significance (*P < 0.05).

## Discussion

The complement system is the important component of both the innate and adaptive immune systems as well as plays an important role in various diseases with acute brain injury, including traumatic brain injury, aneurysmal subarachnoid hemorrhage, ischemic stroke, and hemorrhagic stroke (3). Complement activation is quickly initiated after acute brain injury and subsequently leads to inflammatory cascades and as a consequence, induces a secondary brain injury which is characterized by neuronal death, cerebral edema, and damaged permeability of the blood-brain barrier (15). Overall, excessive activation of the complement system is detrimental to the recovery of neurological function (16).

The protein C1q plays its important role in the immune system and non-immune system (17). It can help to clear microbes and cellular debris from the body and also can participate in the elimination of immature synapses during development of the visual system, neurodegeneration, and cognitive functional formation (18–20). Experimental data showed that C1q was located in microglia and neurons (6, 7). C1q was found to be greatly expressed, not only in animal brain tissues after acute ischemic insult (8), but also in human brain tissues after traumatic brain injury (9). Also, C1q functional activity was enhanced significantly in cerebrospinal fluid of rats with acute cerebral ischemia (8). Interestingly, serum C1q levels were significantly elevated in patients after acute ischemic stroke (10, 11) or traumatic brain injury (12). In the current study, ICH patients had significantly elevated plasma C1q levels as compared with healthy controls. Collectively, C1q in the peripheral blood of patients with ICH is deduced to be, at least in large part, derived from injured brain tissues.

The role of C1q in neuroinflammation has been fully studied. In an experiment of the effect of C1q on release of inflammatory cytokines from cultured microglial cells, extrinsic C1q could obviously lead to the increased secretion of interleukin-6, tumor necrosis factor-alpha and nitric oxide (21). Intriguingly, another study of cultured microglial cells showed the converse results that C1q could markedly depress the lipopolysaccharide-induced production of the preceding inflammatory cytokines and increase monocyte chemoattractant protein-1 levels (22). Similarly, there are two experiments demonstrating the inconsistent data. In a study of neonatal mice with hypoxic-ischemic brain injury, C1q-deficiency may exert a neuroprotective effect (23). Alternatively, after middle cerebral artery occlusion in mice, the complement inhibitor soluble complement receptor-1 markedly decreased cerebral infarct volume and improved neurologic outcome, presumably by blocking the downstream effects of postischemic cerebral C1q expression (24). Clearly, function of C1q is not limited to the neuroinflammation. C1q may be helpful for synaptic circuit refinement during developmental stages and adult plasticity (20). In addition, after mild brain injury, C1q may mediate sleep spindle loss following epileptic spikes (25). Overall, C1q must play a very crucial role in acute brain injury and nevertheless it may function as a double-edge sword.

There have been two epidemiological investigations showing the relation of circulating C1q levels to the severity of ischemic stroke in humans (10, 11). A clinical study demonstrated that patients displayed a significantly higher serum C1q level after acute ischemic stroke than healthy subjects and moreover, serum C1q levels were substantially and positively correlated with the maximum diameter of infarction volume (10). Similarly, in patients with acute ischemic stroke, raised serum C1q levels were significantly and positively related to the National Institute of Health Stroke Scale and the diameter of maximum transverse section based on diffusion-weighted imaging of brain magnetic resonance imaging, indicating that serum C1q may be in close correlation with severity of neurological impairment and infarct volume (11). Nevertheless, in two previous clinical studies regarding human acute ischemic stroke, neurological function was not assessed and no multivariate analysis of note was performed (10, 11) The valuable findings were that (1) a significant elevation of serum C1q levels existed in patients, as compared to healthy individuals; (2) serum C1q levels were independently correlated with clinical and radiological severity, which were reflected by GCS score and Rotterdam computed tomography classification; and (3) serum C1q appeared as an independent predictor for a poor 6 month post-injury outcome (extended GOS score of 1-4) (12). Such data indicate that circulating C1q may be a biomarker of acute brain injury.

As far as we know, it is unclear whether there is a correlation between C1q levels in peripheral blood and severity plus clinical outcomes following ICH. This study not only demonstrated that ICH patients had significantly higher plasma C1q levels than healthy controls, but also showed that plasma C1q levels were independently correlated with hemorrhagic severity assessed *via* GCS score and hematoma volume, as well as independently predicted the development of a poor outcome 3 months after hemorrhage. Notably, there were not statistical differences in AUC for assessing prognostic ability between plasma C1q levels and GCS score plus hematoma volume. Taken together, plasma C1q levels may be intimately related to hemorrhagic severity and functional outcome after ICH, substantializing plasma C1q as a potential prognostic biomarker of ICH.

Two limitations warrant to be mentioned in the current study. First, we performed a single-center prospective cohort study of 101 patients with ICH. Although this patient number is enough for statistical analysis *via* calculation of sample size, it is of clinical value that such conclusions can be validated in a larger cohort study. Second, plasma C1q levels were measured in a time point after ICH in this study and therefore dynamic change of plasma C1q levels is not discovered. Investigating serial change of its level in future may be of clinical significance.

## Conclusions

As far as we know, this is a first series for measuring plasma C1q levels after acute ICH and, thereby found some interesting results: (1) ICH patients have significantly higher plasma C1q levels than healthy individuals, (2) plasma C1q levels are independently correlated with GCS score and hematoma volume, (3) plasma C1q levels independently predict 3-month post-stroke poor prognosis, and (4) plasma C1q levels exhibit significant prognostic ability under ROC curve. Thus, plasma C1q levels may be highly correlated with hemorrhagic severity and could accurately reflect functional outcome after ICH, indicating that C1q may participate in pathophysiological processes underlying acute brain injury and plasma C1q may be selected as a potential prognostic predictor for ICH.

## Data Availability Statement

The raw data supporting the conclusions of this article will be made available by the authors, without undue reservation.

## Ethics Statement

The studies involving human participants were reviewed and approved by Affiliated Hangzhou First People’s Hospital, Zhejiang University School of Medicine (Opinion number: [2020] Medical Ethics Review No. (058)-01). The patients/participants provided their written informed consent to participate in this study.

## Author Contributions

All authors listed have made substantial, direct, and intellectual contribution to the work and approved it for publication.

## Funding

This work is financially supported by key research and development projects of Zhejiang province (Grant Number 2020C03071), the construction fund of medical key disciplines of Hangzhou (Grant Number OO20200485, OO20200055).

## Conflict of Interest

The authors declare that the research was conducted in the absence of any commercial or financial relationships that could be construed as a potential conflict of interest.

## Publisher’s Note

All claims expressed in this article are solely those of the authors and do not necessarily represent those of their affiliated organizations, or those of the publisher, the editors and the reviewers. Any product that may be evaluated in this article, or claim that may be made by its manufacturer, is not guaranteed or endorsed by the publisher.

## Abbreviations

GCS, Glasgow Coma Scale; ICH, intracerebral hemorrhage; ROC, receiver operating characteristic; AUC, area under curve; GOS, Glasgow Outcome Scale; OR, odds ratio; 95% CI, 95% confidence interval; Complement component 1q, C1q.
